# Destruction of the brush border by *Salmonella enterica* sv. Typhimurium subverts resorption by polarized epithelial cells

**DOI:** 10.3389/fmicb.2024.1329798

**Published:** 2024-06-04

**Authors:** Alfonso Felipe-López, Nicole Hansmeier, Michael Hensel

**Affiliations:** ^1^Abt. Mikrobiologie, Universität Osnabrück, Osnabrück, Germany; ^2^CellNanOs—Center of Cellular Nanoanalytics Osnabrück, Universität Osnabrück, Osnabrück, Germany

**Keywords:** epithelial cells, brush border, endocytosis, ezrin, invasion

## Abstract

*Salmonella enterica* serovar Typhimurium is an invasive, facultative intracellular gastrointestinal pathogen that destroys the brush border of polarized epithelial cells (PEC). The brush border is critical for the functions of PEC because it resorbs nutrients from the intestinal lumen and builds a physical barrier to infecting pathogens. The manipuation of PEC during infection by *Salmonella* was investigated by live-cell imaging and ultrastructural analysed of the brush border. We demonstrate that the destruction of the brush border by *Salmonella* significantly reduces the resorption surface of PEC along with the abrogation of endocytosis at the apical side of PEC. Both these changes in the physiology of PEC were associated with the translocation of type III secretion system effector protein SopE. Additionally, the F-actin polymerization rate at the apical side of PEC was highly altered by SopE, indicating that reduced endocytosis observed in infected PEC is related to the manipulation of F-actin polymerization mediated by SopE and, to a lesser extent, by effectors SopE2 or SipA. We further observed that in the absence of SopE, *Salmonella* effaced microvilli and induced reticular F-actin by bacterial accumulation during prolonged infection periods. In contrast to strains translocating SopE, strains lacking SopE did not alter resorption by PEC. Finally, we observed that after engulfment of *Salmonella*, ezrin was lost from the apical side of PEC and found later in early endosomes containing *Salmonella*. Our observations suggest that the destruction of the brush border by *Salmonella* may contribute to the pathogenesis of diarrhea.

## Introduction

The intestine is the main organ of the digestive tract and is responsible for the digestion and resorption of nutrients from the diet. The intestinal tract has evolved a specialized surface formed predominantly by polarized epithelial cells (PEC) to fulfill its function. Extensions of the intestinal epithelium are designated as villi. At the cellular level, each PEC of the epithelial layer develops at its apical surface actin-based membranous protrusions called microvilli (MV). MV are the functional structures of epithelial cells for nutrient resorption. Two morpho-physiological features contribute to this function: (a) MV augment the functional surface for resorption since cells possess a high number of MV (>1,000 MV), and (b) each microvillus contributes to the secretion of digestive enzymes such as disaccharidases and peptidases (McConnell and Tyska, 2007; McConnell et al., 2009; Revenu et al., 2012). The biochemical activity of MV is dependent on the function of structural proteins specifically localized in each microvillus. Loss of actin-coordinating proteins such as epsin, villin, or fimbrin (plastin I) by gene knockout negatively affects the localization and secretion of digestive enzymes (Revenu et al., 2012). Therefore, the membrane protrusions by MV are only functional if digestive enzymes can be properly secreted to the external milieu (Delacour et al., 2016).

MV also contribute to regulating the microbiota that reside along the intestine. Nevertheless, pathogenic bacteria such as enteropathogenic *Escherichia coli* (EPEC), enterohaemorrhagic *E. coli* (EHEC), enterotoxigenic *E. coli* (ETEC), *Helicobacter pylori*, or *Salmonella enterica* can disturb the integrity and function of the intestinal epithelium. After adhesion to host cells, these pathogens efface MV by translocation of virulence proteins that modify (a) actin, (b) increase the adhesion surface, in the case of EPEC and *H*. *pylori*, or (c) alter the transporter expression and localization by ribosylating toxins as the labile toxin (LT) of ETEC (Segal et al., 1996; Tan et al., 2009; Wong et al., 2011; Sheikh et al., 2022; Felipe-Lopez et al., 2023).

MV effacement results from the remodeling of actin and leads to the formation of pedestals, a typical feature observed during infection by EPEC or EHEC (Frankel et al., 1998; Wong et al., 2011; Lai et al., 2013). Additionally, brush border destruction is commonly accompanied by the formation of microcolonies at the host cell's apical side (Pedersen et al., 2017). This pathogenic interference leads to a loss of resorption by PEC (Dean et al., 2006) since digestive enzymes and the cotransporter Na^+^/Glucose SGLT-1 are also relocated to pedestals. Reduction of the resorption ability of enterocytes is also associated with high doses of infection and with the translocation of effector proteins such as Map, Tir, EspF, and the adhesin Intimin. In all these observations, EPEC formed microcolonies at the apical side of PEC. EPEC effector proteins NleH specifically target EPS8, hindering its function in actin bundling for pedestal formation (Dean et al., 2006). Infections by *H. pylori* alter the uptake of iron by PEC (Tan et al., 2011), as *H. pylori* does not diminish the uptake but increases the resorption of transferrin. Recruitment of the transferrin receptor to microcolonies at host cell apical side was dependent on effector protein CagA. In contrast to EPEC/EHEC and *H. pylori, S. enterica* serovar Typhimurium (STM) is an invasive pathogen that destroys the brush border of PEC *in vivo* and *in vitro* (Takeuchi, 1967; Finlay et al., 1988; Ginocchio et al., 1992; Felipe-Lopez et al., 2023). The SPI1-encoded type III secretion system (SPI1-T3SS) translocates a cocktail of effector proteins into host cells after intimate contact. Collectively, these effector proteins remodel the actin cytoskeleton of host cells, induce MV effacement, and internalize STM. Translocation of effector proteins also induces proinflammatory responses in intestinal PEC [Galan, 2021; reviewed in Fattinger et al. (2021b)].

The invasion of enterocytes by STM is highly cooperative and allows the invasion of multiple bacteria in the same infection foci (Lorkowski et al., 2014; Felipe-Lopez et al., 2023). The invasion concludes with extensive reorganization of the brush border and the formation of a structure called reticular F-actin (RA) at the apical side of PEC. Previous observations of STM-induced alterations of the apical site of PEC and the F-actin cytoskeleton are compiled in Supplementary Figure 1, demonstrating the ultrastructural features of RA. Fine filaments arose from the invasion foci to the periphery of the apical side of the cells once bacteria were internalized (Felipe-Lopez et al., 2023). In this experimental setup, the formation of RA on PEC was dependent upon the translocation of SopE and observed in MDCK and Caco-2 BBe1 cells but not in non-polarized cells, e.g., epithelioid cell line HeLa. RA remained even after bacterial internalization, coinciding with reduced recovery of MV structures as demonstrated by correlative confocal laser-scanning microscopy (CLSM) and atomic force microscopy (AFM) or scanning electron microscopy (SEM) (Kommnick et al., 2019; Felipe-Lopez et al., 2023). The internalization of STM, formation of RA, and disruption of the brush border might, therefore, alter the physiological apical resorption of PEC. Indeed, a recent study revealed that infection by STM altered the expression of ascorbic acid transporters in mice due to the inflammatory response caused by SPI1-T3SS effector proteins. However, no structural modifications of the brush border or enterocytes were mentioned (Teafatiller et al., 2023).

In this study, we investigate the physiological consequences of the translocation of SPI1-T3SS effector proteins. We focused on SopE and SopE2, which act as guanidine exchange factors for Rac1 and Cdc42 in mammalian host cells (Friebel et al., 2001). We show that RA is induced either by an invasion of STM expressing *sopE* or by the accumulation of STM devoid of *sopE* over time. The destruction of brush border architecture also demonstrated that resorption by PEC was disturbed after MV effacement and internalization of STM, which was dependent on SopE. The loss of MV by strains lacking SopE was insufficient to fully abrogate the uptake of fluid tracers by infected cells. We observed that STM invasion was accompanied by delocalization of ezrin from the apical side of host cells. Ezrin links F-actin to the plasma membrane of MV, and the interaction of ezrin with F-actin is controlled by the phosphocycling of ezrin [reviewed in Pelaseyed and Bretscher (2018)]. Therefore, we propose that as a consequence of the delocalization of ezrin and the resulting actin reorganization on the apical side of PEC, endocytosis of PEC is highly altered. The loss of MV and formation of RA during STM invasion thus may contribute to the pathogenesis of diarrhea during Salmonellosis.

## Results

### Role of STM effector SopE in invasion of polarized epithelial cells, microcolony formation, and induction of reticular actin

Our prior work indicated a key role of SPI1-T3SS effector protein SopE in manipulating the apical side of PEC (Felipe-Lopez et al., 2023). However, the majority of clinical isolates of STM lack SopE, while all strains harbor SopE2 (Mirold et al., 1999). As STM strains lacking SopE also cause self-limited intestinal infections, in this study, we aim to analyze the effects of STM with or without SopE on PEC function and epithelial integrity.

**Figure 1:**
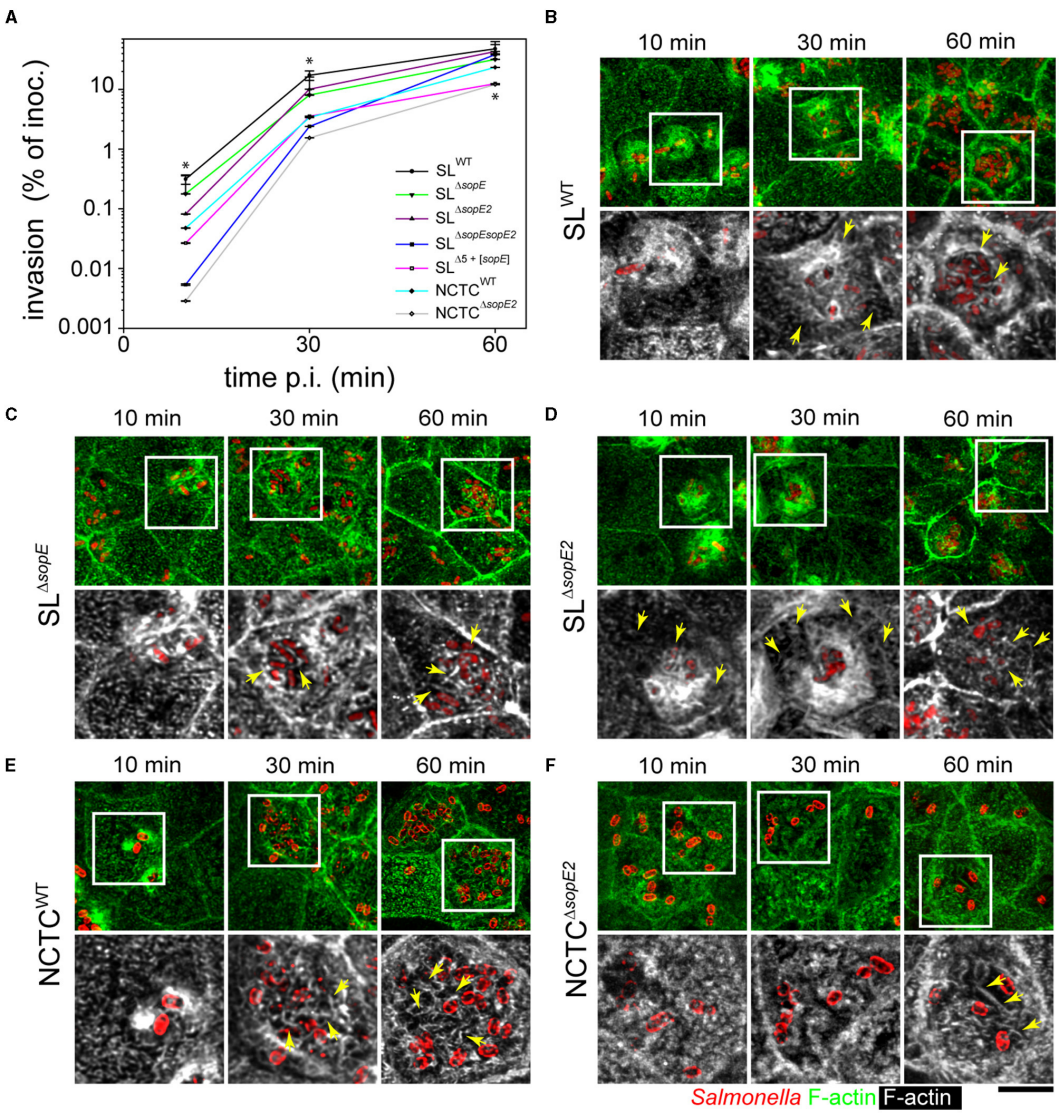
Effector proteins SopE and SopE2 are required for the manipulation of apical F-actin of PEC by STM. **(A)** Polarized MDCK monolayers were infected with STM WT and various mutant strains lacking *sopE* or *sopE* for 10, 30, or 60 min as indicated. After washing, non-internalized bacteria were killed by incubation with a medium containing 100 μg × ml^−1^ gentamicin for 1 h. Cells were lysed and internalized STM was quantified by plating serial dilutions of lysates for CFU determination. Invasion is expressed as the percentage of internalized bacteria of the inoculum applied. One-way ANOVA was applied for statistical analysis, and the results are indicated as; ^*^p < 0.05. **(B–F)** STM infection leads to MV effacement and formation of microcolonies at the apical side of PEC. MDCK cells were infected with STM wild-type (WT) or mutant strains expressing mTagRFP **(B–D)** as fluorescently labeled, or WT and mutant strains immuno-stained with O-antigen of LPS **(E, F)**. Samples were fixed at 10, 30, or 60 min p.i., labeled with Phalloidin-Alexa488 (green in overview, white in details), and analyzed by confocal laser-scanning microscopy (CLSM). STM SL strains harbored pFPV-mCherry (red) for detection in **(B–D)**, while STM NCTC strains were immunolabeled for O-antigen (red) in **(E, F)**. Micrographs show accumulation of STM and alteration of F-actin at various time points p.i. Yellow arrows indicate RA along the apical side of MDCK cells and adjacent to STM cells. Scale bar, 10 μm (overviews), 5 μm (details).

The cell line Madin-Darby Canine Kidney (MDCK) was used as PEC for infection experiments and to quantify invasion of PEC by wild-type (WT) strains SL1344 harboring both *sopE* and *sopE2* (SL^WT^) and NCTC12023 harboring only *sopE2* (NCTC^WT^). Isogenic *sopE*/*sopE2*-deficient or *sopE2*-deficient mutant strains were performed (Figure 1A). At 10 and 30 min p.i., the invasion by SL^Δ*sopEE2*^and NCTC^Δ*sopE2*^ strains was about 100- and 10-fold, respectively, lower than that of corresponding WT strains. Although invasion by STM SL^Δ*sopE*^, SL^Δ*sopE2*^, or SL^Δ*sopE*^ ^*sopE*2^ was lower at earlier time points, these strains reached levels similar to SL^WT^ at 60 min p.i. (Figure 1A). Invasion by SL^Δ*sopE*^ and NCTC^WT^, which naturally lacks SopE, was slightly but significantly lower than that of SL^WT^. Using a reductionist approach (Felipe-Lopez et al., 2023) with a strain lacking *sipA sopABEE2* (SL^Δ5^) with similar invasion to an *invF* mutant strain, we found that STM SL^Δ5+[*sopE*]^ also showed augmented invasiveness; however, this was still 10-fold less than STM SL^WT^ and was similar to that quantified for STM NCTC^Δ*sopE2*^.

We previously demonstrated that MDCK and Caco-2 Bbe1 (C2BBe1) cells form homogeneous monolayers, and with such a model, epithelia infection of the apical side by STM can be analyzed. MDCK cells were infected by adding STM inoculum to the apical reservoir as described before (Hölzer and Hensel, 2012; Felipe-Lopez et al., 2023), and imaging was performed by CLSM. Micrographs of infected MDCK cells registered at the same time points demonstrated that only SL1344 background strains harboring SopE triggered membrane ruffling and MV effacement at 10 min p.i. (Figures 1B, D). Additionally, while MDCK cells infected for 30 min with STM SL^WT^ or SL^Δ*sopE2*^showed RA distributed on the apical side of PEC (Figures 1B, D), STM SL^Δ*sopE*^did not induce large ruffles and bacteria accumulated at the apical side of the cell in microcolonies (Figure 1C). Cells infected by STM SL^Δ*sopE*^showed RA only adjacent to bacteria but not extending further on the apical side (Figure 1B vs. Figure 1C). At 60 min p.i., RA was present only in cells highly infected by any of the strains tested (Figures 1B–D). In contrast to strains with SL1344 background, STM NCTC^WT^ only induced moderate membrane ruffling at 10 min p.i. (Figure 1E). STM NCTC^Δ*sopE2*^ triggered a low accumulation of actin around the bacterial cells, but it was not accompanied by membrane extensions (Figure 1F). At 30 min p.i., STM NCTC^WT^ accumulated in microcolonies at the apical side of cells, similar to STM SL^Δ*sopE*^(Figure 1C vs. Figure 1E), and RA surrounded the bacteria, which was not evenly distributed on the cell, compared to SL^WT^ strain (Figure 1B vs. Figure 1E). MDCK cells infected by STM NCTC^Δ*sopE2*^only showed small changes in the actin cytoskeleton (Figure 1F) associated with four to six bacteria, but no bacterial microcolonies were observed for this strain. At 60 min p.i., the STM NCTC^WT^ strain caused a complete loss of the brush border architecture (Figure 1E). Bacteria were grouped in microcolonies, and RA was tightly associated with bacterial cells. Only slight reorganization of the actin cytoskeleton was appreciable in cells infected by NCTC^Δ*sopE2*^, but on cells infected by this strain, microcolonies were absent, and only short filaments of actin were observed around invading bacteria (Figure 1F). RA was evenly distributed at the apical side of host cells infected by STM SL^WT^ at early time points (10 min p.i., Figure 1B), whereas in cells infected by STM NCTC^WT^, RA was tightly associated with invading bacteria and only fully visible at 60 min p.i. (Figure 1E).

### Role of SPI1-T3SS effector proteins in breaching epithelial barriers

**Figure 2:**
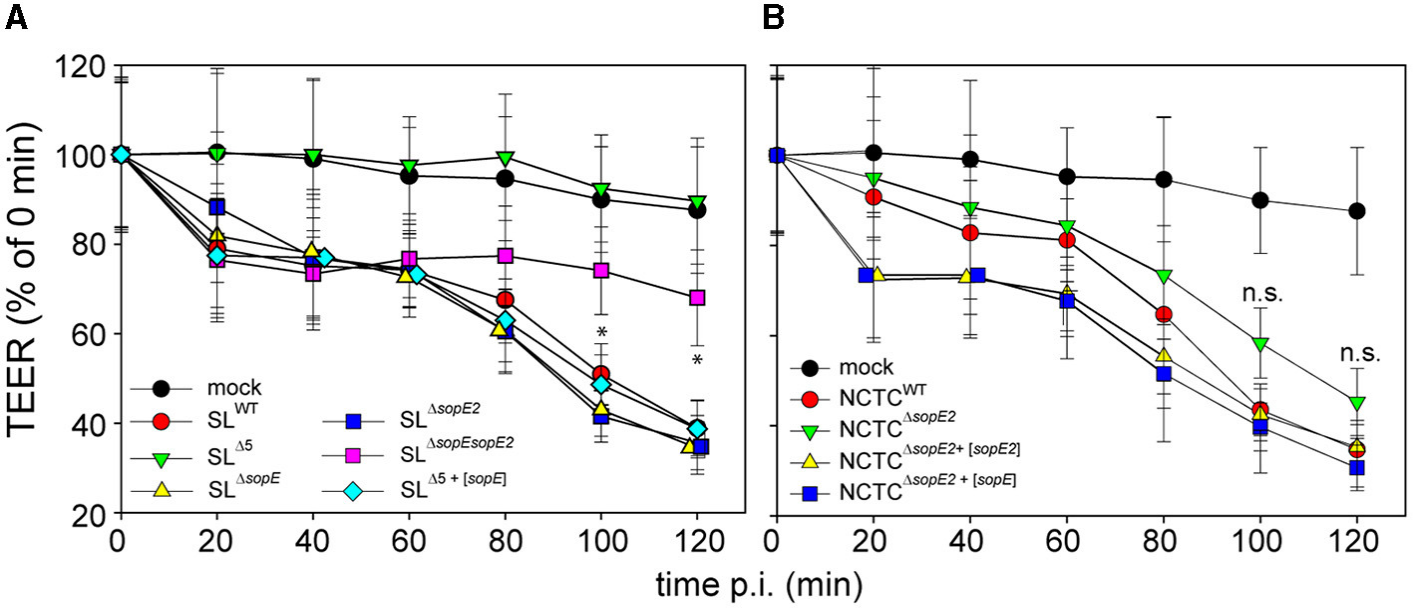
Effector proteins SopE and SopE2 are key factors for the disruption of the epithelial barrier function of PEC by STM. C2BBe1 cells were seeded on transwell inserts with polycarbonate filters of 0.4 μm pore size and cultured for 10–15 d to allow the formation of polarized monolayers. Polarization is indicated by the increase of transepithelial electrical resistance (TEER) to 500–700 Ω per well. Polarized monolayers were infected at MOI 25 with STM WT or various mutant strains lacking *sopE, sopE2*, or *sipA sopA sopB sopE sopE2* (strain Δ5), without or with plasmids harboring *sopE* or *sopE2*. Infections were performed with isogenic STM strains in strain background SL1344 **(A)** or NCTC12023 **(B)**. The TEER was scored over a period of 2 h p.i. The TEER is expressed as the percentage of TEER of respective transwells determined immediately prior to infection. Means and standard deviations are shown for TEER quantifications. All experiments were repeated at least three times with two technical replicates. One-way ANOVA was applied for statistical analysis, and the results are indicated as not significant (n.s.); *p < 0.05.

We sought to further characterize the effects of SopE on PEC physiology and deployed the human colonic epithelial cell line C2BBe1. Polarization of these cells leads to epithelial layers with high transepithelial electrical resistance (TEER), and pathogenic manipulation of PEC decreases TEER. The TEER of C2BBe1 polarized monolayers was monitored during infection by STM (Figures 2A, B). As previously reported (Felipe-Lopez et al., 2023), deletion of only *sopE* had no effect on the destruction of the epithelial barrier. On the contrary, deletion of both *sopE* and *sopE2* in SL1344 partially ablated the epithelial damage since at 40 min p.i., the TEER dropped to ca. 70% of the value prior to infection and remained low. Only after 120 min p.i. was a further slight decay observed. Infection by STM SL^Δ5+[*sopE*]^ caused decay of TEER similar to that observed in infection by STM SL^WT^ (Figure 2A).

The TEER of cells infected by NCTC^WT^ dropped slower than that of cells infected by STM SL^WT^ (Figure 2B). At 60 min p.i., STM NCTC^WT^ infection decreased TEER to 85% of TEER prior to infection, while the TEER of SL^WT^-infected cells decreased to 75% already 20 min p.i. TEER decay of cells infected by STM NCTC^Δ*sopE2*^ was slower than for cells infected by NCTC^WT^ (Figure 2A vs. Figure 2B) and decayed in a rather linear manner (Figure 2A). Since the effects observed were associated with effector proteins SopE or SopE2, NCTC^Δ*sopE2*^ was complemented with *sopE* from SL1344. This strain caused the rapid decay of TEER identical to the phenotype induced by infection with STM SL^WT^ (Figure 2B).

These data indicate that *Salmonella's* alteration of the epithelial barrier function is primarily dependent on either single translocation of SopE (see SL^Δ5+[*sopE*]^) or the translocation of a group of effector proteins controlling F-actin such as SipA, SopE2 and, to a lesser extent, SopB, when SopE is absent.

### F-actin remodeling by STM induces the formation of reticular actin

**Figure 3:**
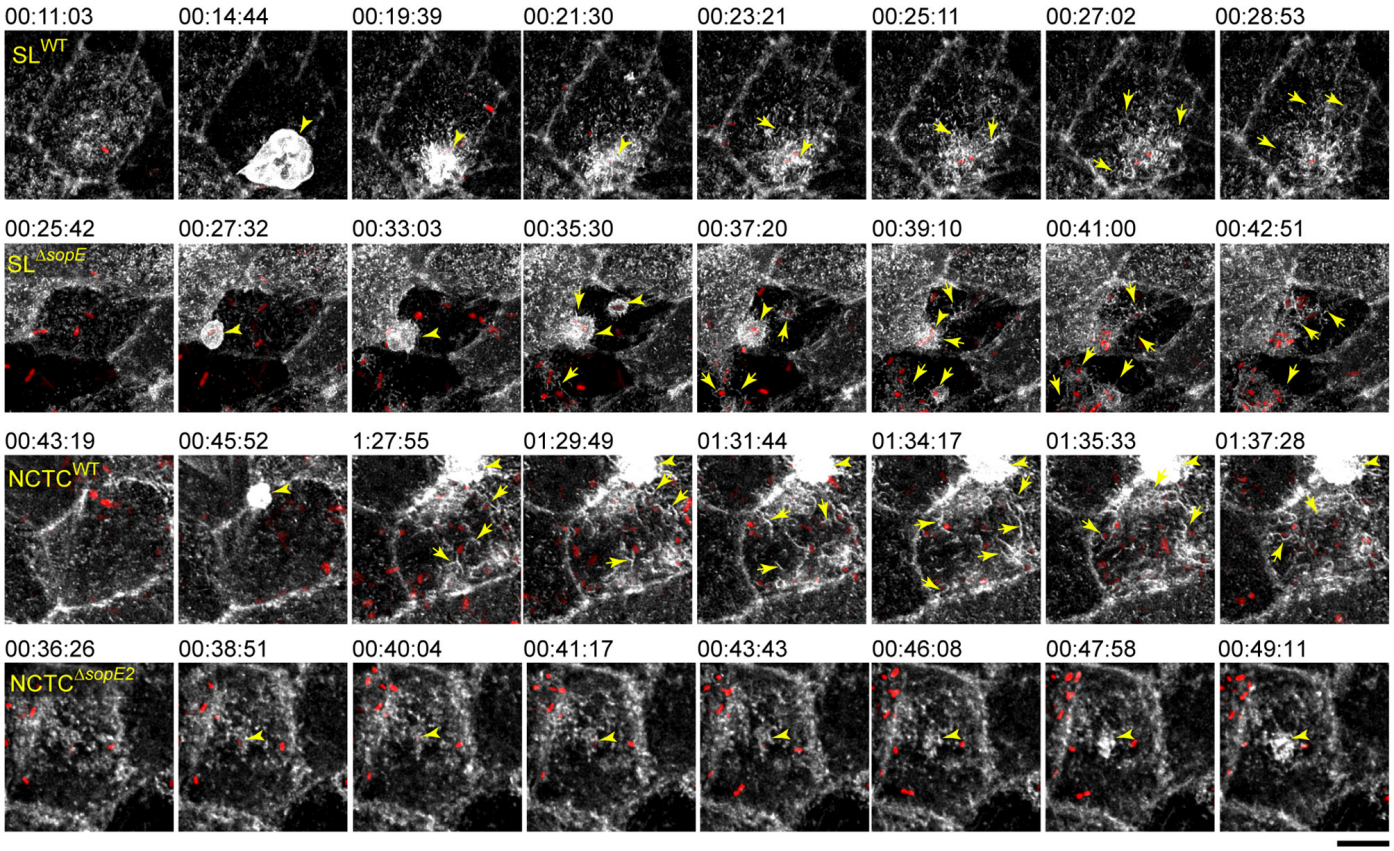
Brush border architecture is destroyed by bacterial cells clustering on the apical side of PEC and substituted with RA. MDCK cells expressing Lifeact-eGFP were grown in fluoro dishes for 5 d. Lifeact-eGFP labels F-actin filaments in living cells, which are shown in white. PEC were infected with various STM strains as indicated, and image acquisition by SD with maximal acquisition speed (2–3 frames per min) was started immediately after infection for 120 min of acquisition. Maximum intensity projection (MIP) images show the dynamics of STM accumulation at the apical side of MDCK cells, MV effacement, and formation of RA. Arrowheads indicate F-actin accumulation in membrane ruffles induced by STM. Arrows indicate the formation of RA. Scale bar, 15 μm. Timestamp, h:min:sec. See Supplementary Movie 1 for the time-lapse series.

Our observations from fixed time points suggested that SopE not only enhanced invasiveness but also strongly influenced actin reorganization after the full engulfment of STM and the events after the invasion. The RA already appeared after 10 min in cells infected by STM SL^WT^ but was delayed to 60 min p.i. in infections by STM SL^Δ*sopE*^ or STM NCTC^WT^. To investigate the formation of RA by apparently distinct mechanisms, the infection of MDCK LifeAct-eGFP cells by various STM strains was followed by live-cell imaging (LCI). The infection of STM SL^WT^ triggered ruffle formation within 8 min after initial apical adhesion (Figure 3; Supplementary Movie 1), as previously reported (Felipe-Lopez et al., 2023). After full engulfment of STM SL^WT^ and ruffle retraction, thin filaments of actin appeared surrounding the invasion locus on the apical side. These filaments gradually increased over the next 10 min from the periphery to the invasion locus, forming a reticular F-actin structure (Figure 3, SL^WT^, Supplementary Movie 1). In contrast, STM SL^Δ*sopE*^induced only small ruffles, and changes in brush border architecture were not detected when a single bacterium invaded (Figure 3, SL^Δ*sopE*^). However, when the same cell was subsequently invaded by further bacteria, the brush border was lost, and RA appeared between the invading bacteria (yellow arrows). F-actin filaments only emerged close to the invasion foci but not from the periphery, as observed for STM SL^WT^ (Figure 3). Further invasion events increased the formation of RA, which was inter-connected to the previous invasive bacteria. STM SL^Δ*sopE2*^ showed similar behavior as STM SL^WT^ (data not shown).

STM NCTC^WT^ induced RA similar to STM SL^Δ*sopE*^ (Figure 3, NCTC^WT^ yellow arrows), but this phenotype was delayed. After the first invasion event, other bacteria induced the formation of RA and brush border effacement. These bacteria were surrounded by intense actin signals. Actin filaments were distributed mainly at the periphery and around invading STM. The deletion of *sopE2* in STM NCTC altered the ability of *Salmonella* to induce RA (Figure 3, NCTC^Δ*sopE2*^). Cells infected by this strain also formed small clusters and showed further brush border destruction; however, there was no induction of RA even after several bacteria had invaded. Only short and very thin F-actin filaments were locally associated with bacteria.

These results demonstrate that while SopE was sufficient to cause MV effacement and formation of RA by one single bacterial cell, for strains lacking SopE infection over long time periods, the accumulation of multiple bacteria at the apical side was necessary to cause similar alterations to the host cell. Strains lacking both *sopE* and *sopE2* were unable to induce the formation of RA, and we conclude that RA is dependent on SopE and/or SopE2.

### Intracellular STM affects brush border regeneration

**Figure 4:**
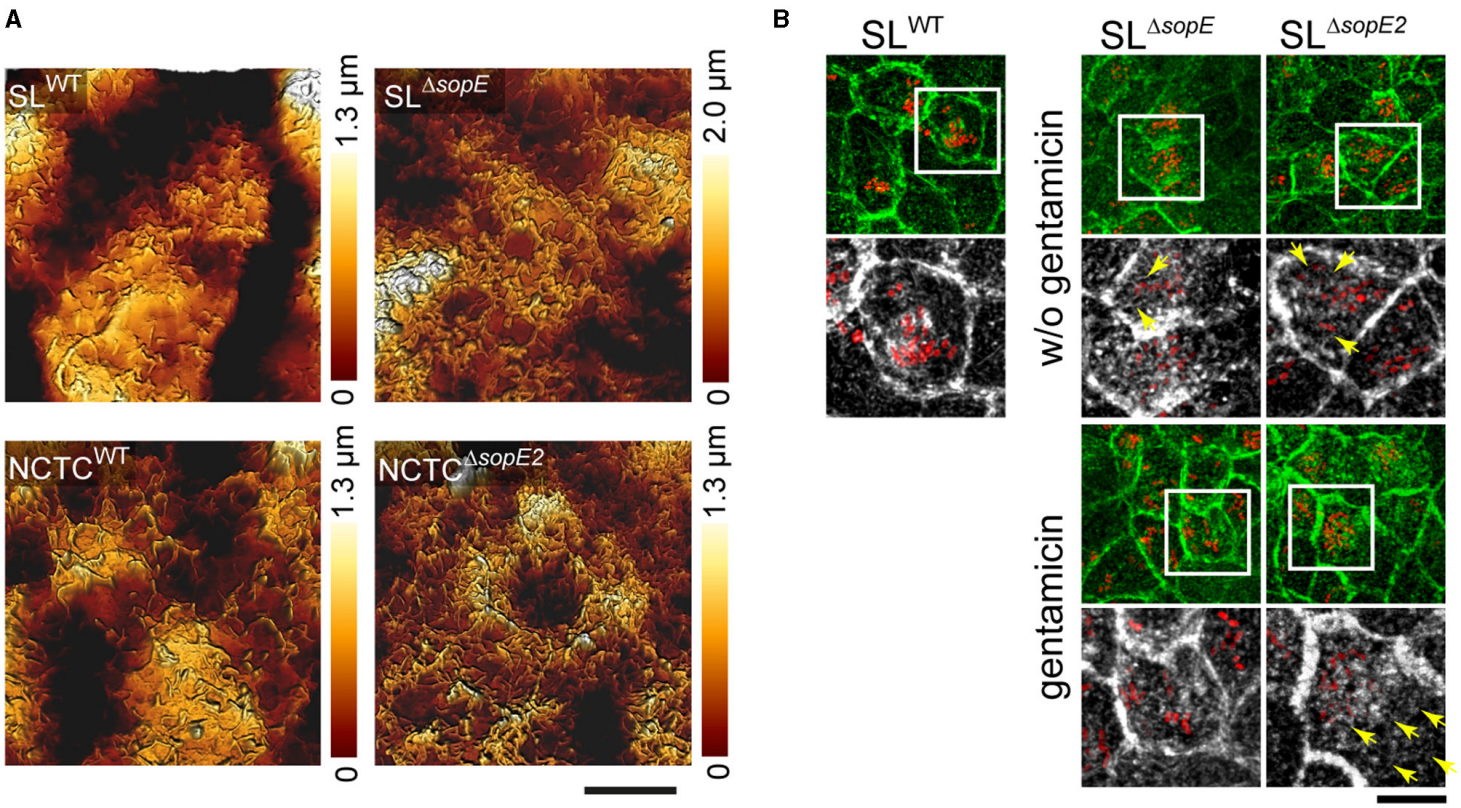
Brush border architecture is not restored in PEC infected by STM strains harboring *sopE*. Polarized MDCK cells expressing Lifeact-eGFP were grown as in Figure 3 and were infected with *Salmonella* WT for 25 min. F-actin is shown in green. Non-internalized STM were removed by washing, and cells were incubated for 1 h in media without gentamicin or in a medium containing 100 μg × ml^−1^ gentamicin to kill the remaining extracellular bacteria. Cells were fixed and stained as indicated in Figure 1 and observed by CLSM. **(A)** 3D topology micrographs show the degeneration of the apical side of MDCK cells after internalization of *Salmonella* (cells only treated with gentamicin). **(B)** Formation of RA is induced by SopE. Extracellular STM were removed by washing, and cells were incubated with or without the addition of gentamicin for 1 h. Only cells infected by SL1344^Δ*sopE*^ but not treated with gentamicin showed RA. Yellow arrows indicate the formation of RA after internalization of STM at various time points of infection. Scale bars, **(A)** 5 μm, **(B)** 20 μm, and 10 μm for overview and detail, respectively.

Our data demonstrate that RA only appeared shortly after the engulfment of *sopE*-harboring STM or after the accumulation of multiple *sopE*-deficient STM. Therefore, we tested if invasion only controls the formation of RA and loss of the brush border or if intracellular *Salmonella* also interferes with the regeneration of the brush border and the disappearance of RA. MDCK cells were infected by various strains lacking SopE or SopE2 in either strain background for 30 min. Infected cells were then washed and treated with gentamicin for 1 h or only rigorously washed and further incubated for 1 h w/o gentamicin addition. Micrographs generated by AFM (Figure 4A) or CLSM (Figure 4B) show that even after gentamicin treatment to kill non-internalized STM, RA was still present in cells infected by STM SL^WT^ and, to a lesser extent, STM NCTC^WT^. Cells infected by STM NCTC^Δ*sopE2*^ or STM SL^Δ*sopE*^ showed a slight recovery of brush border after gentamicin treatment (Figures 4A, B). Additionally, F-actin structures were concentrated in clusters of intracellular bacteria (Figure 4B). Cells infected by *sopE-*deficient strains also showed recovery of MV. Interestingly, cells infected by SL^Δ*sopE*^ without gentamicin treatment still possessed RA, and as observed for STM SL^WT^, bacteria were surrounded by actin. Altogether, these results show that STM continues to alter the brush border architecture after internalization.

### Reticular actin displaces resorptive areas, and SopE alters actin polymerization at the apical side of host cells

**Figure 5:**
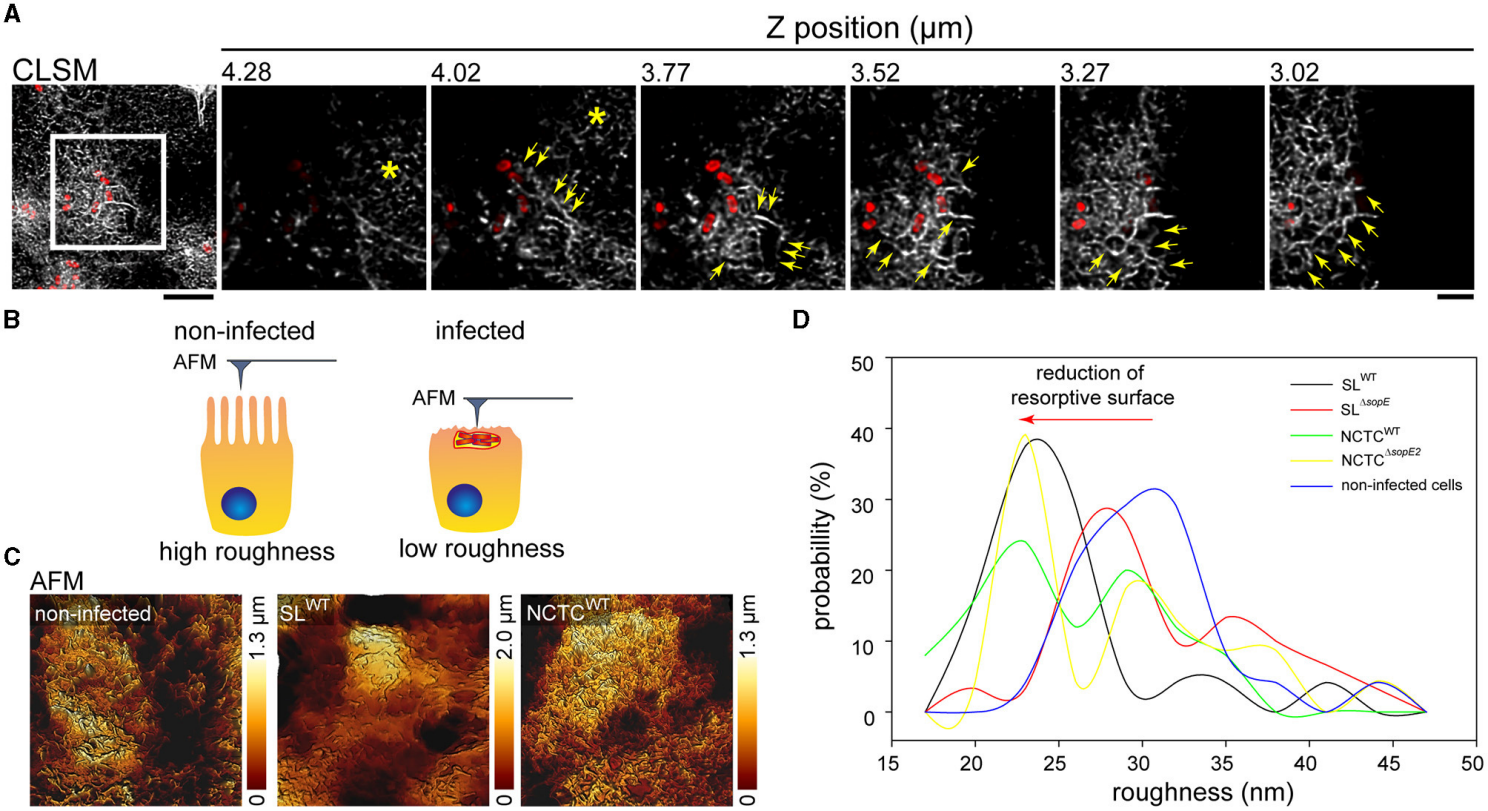
Destruction of the brush border by *Salmonella* invasion reduces the apical surface. **(A)** MDCK cells were infected with STM SL^WT^ (red), and F-actin was labeled (gray). Representative CLSM image of a group of cells infected by STM (overview). Detailed images from distinct planes along the *Z*-axes show the organization of RA at the apical side of STM-infected cells (yellow arrows) and the remaining intact MV (asterisk). Scale bar, 10 μm (overview) and 5 μm (detail). **(B)** Model of the roughness measurement by AFM. When cells possess MV on the apical surface, the roughness and deepness are higher than those of cells infected by STM, showing MV effacement with a flatter surface and reduced deepness. **(C)** AFM images of the 3D topology of infected cells without the brush border. Cells were prepared as indicated in Figure 1 for AFM scanning and CLSM. **(D)** Roughness of at least 25 cells was determined by Young's module and presented as the probability of the roughest area in the cell.

Our results indicate that SopE and SopE2 induce the formation of RA after brush border effacement. Such gross alteration of the F-actin cytoskeleton might change the physiological properties of the brush border. To address this point, we determined the effect of STM invasion on the roughness of the apical side using AFM (Antonio et al., 2012). Compared to non-infected controls, peaks of roughness probability were shifted to lower roughness in STM-infected cells, in line with the observed loss of brush border (Figure 5). The roughness of the apical side of host cells infected by SL^WT^ was highly reduced compared to non-infected cells (Figures 5C, D). The maximal peak observed in SL^WT^-infected cells was 10 nm less rough than that quantified in non-infected cells (23 vs. 30 nm; Figure 5D). Similarly, STM NCTC^WT^-infected cells showed, on average, a reduced surface roughness compared to non-infected cells (24 vs. 33 nm). However, in contrast to SL^WT^-infected cells, NCTC^WT^-infected cells showed a larger distribution of roughness probabilities. Cells infected by STM SL^Δ*sopE*^ or NCTC^Δ*sopE2*^ showed no reduced roughness but rather values close to the maximal roughness of non-infected cells (28 vs. 30 nm). Cells infected by STM NCTC^WT^ showed, on average, a reduced surface roughness compared to the non-infected cells (24 vs. 30 nm). However, in contrast to SL^WT^-infected cells, NCTC^WT^-infected cells showed a larger distribution of roughness probabilities.

These measurements demonstrate that STM infections fully remodel the apical architecture of PEC, depending on SopE. The reorganization of cells after infection with NCTC background strains naturally lacking SopE was rather moderate, and infected cells also showed high roughness similar to non-infected cells. Based on our LCI results, these cells still contained MV on the apical surface under the experimental conditions applied.

**Figure 6:**
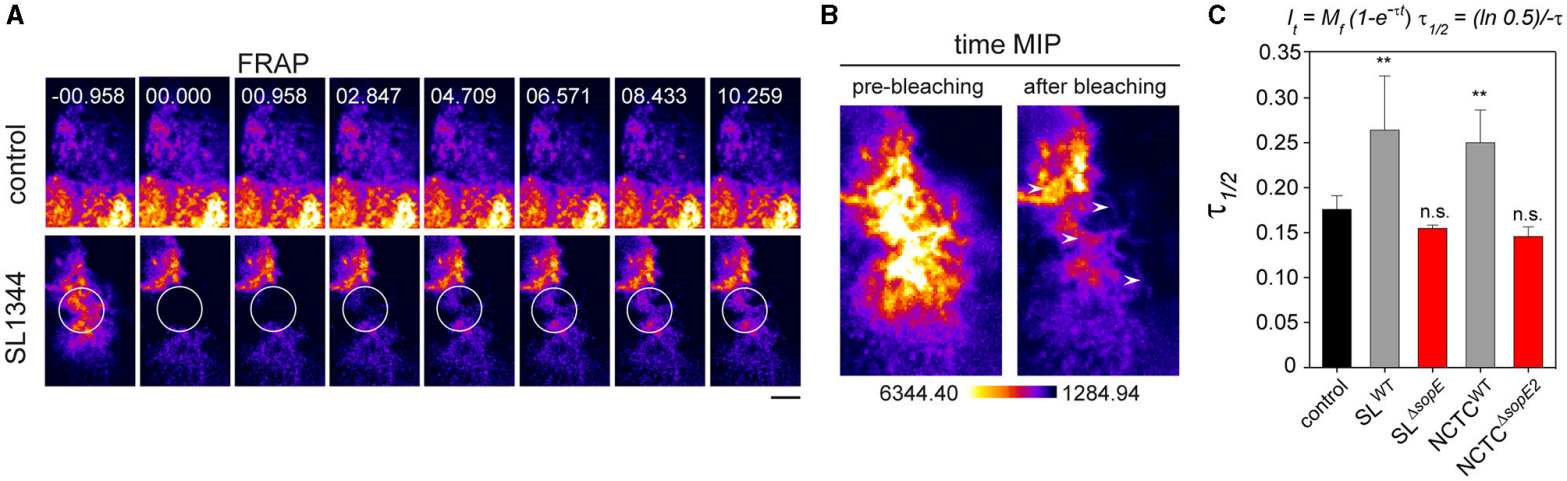
*Salmonella* invasion alters the polymerization of F-actin at the host cell's apical side. PEC were infected at MOI 25 for 25 min and then washed five times with pre-warmed MEM without phenol-red to remove extracellular bacteria. Infected cells in chamber slides were observed by SD microscopy. Areas indicated by circles were photobleached using a laser line 488 nm at 100% transmission for 100 ms. **(A)** Representative images of fluorescence recovery of F-actin after photo-bleaching (FRAP) of STM-infected cells are shown. Non-infected cells without photobleaching served as control. **(B)** Time maximal intensity projections (MIP) of a representative bleached region before and after bleaching. **(C)** The recovery constant (τ) is strongly reduced in cells infected by STM compared to control. Means and standard deviations of at least 25 cells per condition are shown. Quantification and mathematical adjustment were performed as described (Ishikawa-Ankerhold et al., 2012). Scale bar, 2.5 μm. One-way ANOVA was applied for statistical analysis, and results are indicated as not significant (n.s.); **p < 0.01.

The loss of the absorptive surface of PEC, accompanied by the formation of RA, suggests that actin polymerization at the apical side may also be affected. To quantify the turnover of actin from RA, fluorescence recovery after photobleaching (FRAP) was applied to infected cells showing RA. Data were adjusted to the model proposed by Ishikawa-Ankerhold et al. (2012). After a pulse of 500 ms for photobleaching, RA was recovered (Figures 6A, B) in cells infected by STM SL^WT^, indicating that RA formation by F-actin polymerization continued even when STM was intracellular (Figures 6A, B). Further quantification of the signal recovery revealed that actin polymerization at the apical side of host cells was increased after infection by STM SL^WT^ or NCTC^WT^ (Figure 6C). The cells infected by either SL^Δ*sopE*^ or NCTC^Δ*sopE2*^ did not cause any significant reduction of the actin polymerization rate (Figure 6C). Data from this experimental setup confirmed that actin polymerization was altered by STM infection, even when bacteria were located intracellularly, and this exclusively depended on SopE by SL^WT^ and SopE2 in NCTC^WT^. Furthermore, actin remodeling occurring during STM internalization may not be sufficient to alter the physiological functions of PEC.

### SopE disrupts the physiological resorption of polarized epithelial cells

**Figure 7:**
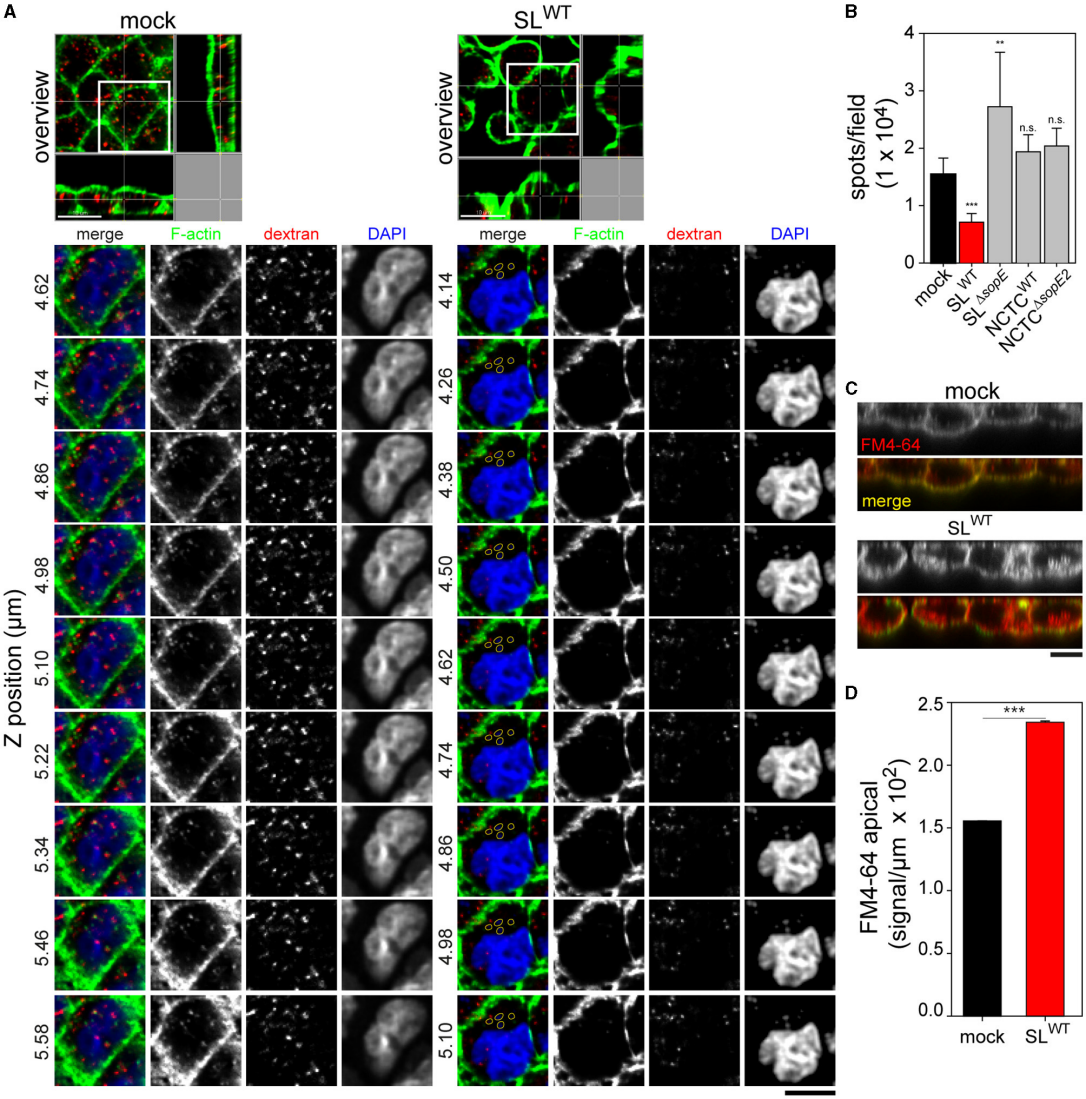
Destruction of the brush border by *Salmonella* disrupts the physiologic uptake of the apical side of polarized epithelial cells. PEC were infected, as described in Figure 1. After gentamicin treatment, cells were pulsed with Alexa568-labeled dextran (red) or membrane dye FM4-64FX (red) for 30 min. Cells were further processed as described in Figure 1; actin was labeled by phalloidin (green), and nuclear and bacterial DNA were stained by DAPI (blue). **(A)** Representative orthogonal images show mock-infected cells or cells infected by STM SL^WT^ cells pulsed with dextran Alexa568. Images show lateral views or the *Z*-axes of the cells containing dextran along the cell body. Various *Z* positions are shown. In infected cells, intracellular STM stained by DAPI are outlined yellow. Scale bars, 10 μm. **(B)** Effect of STM infection on endocytosis of dextran Alexa568. Infection of MDCK cells with various STM strains as indicated or mock infection and dextran Alexa568 pulse were performed as above. The numbers of dextran spots were quantified by Imaris as described in Materials and methods section. **(C)** Representative *Z* projections of cells labeled with membrane stain FM4-64 (red). Cells were mock-infected or infected by STM SL^WT^, and actin (green) was labeled as above. Scale bars, 10 μm. **(D)** Quantification of FM4-64 signal intensities in the apical area of mock-infected or STM SL^WT^-infected MDCK cells. Scale bars, 10 μm. One-way ANOVA was applied for statistical analysis, and the results are not significant (n.s.); **p < 0.01; ***p < 0.001.

Epithelia of the intestine, kidney, and other organs are responsible for the uptake of nutrients and ions. The loss of the brush border architecture abrogates this function. Given that RA is still present even after the complete invasion of STM, we investigated if replacing MV with RA alters the apical resorption of MDCK cells. At 1.5 h p.i., gentamicin-treated cells were pulsed for 30 min with FM4-64 FX to label plasma membrane or with fluid tracer dextran-10,000 Alexa568 to follow endocytic uptake. Cells infected by SL^WT^ internalized less dextran than non-infected cells (Figure 7A). Internalized dextran in cells infected by STM SL^WT^ appeared as small accumulations at the apical side of cells and close to clusters of intracellular bacteria. However, no colocalization was observed between STM and the fluorescent tracer (Figure 7A, detail), indicating that the internalized material was not entering the SCV at this time point of infection. Compared to SL^WT^-infected cells, non-infected cells contained a large number of small-sized vesicles homogenously distributed in the apical region of the cell (Figure 7A). Most of these dextran-positive vesicles colocalized with F-actin, and some were localized at the apical membrane of the cell, indicating recent uptake (Figure 7A, *Z* sections). Such recruitment of actin was not visible in infected cells. Infection by STM SL^Δ*sopE*^ resulted in increased dextran uptake compared to non-infected cells. Infection by NCTC^WT^ or NCTC^Δ*sopE2*^ did not significantly alter the uptake of dextran, and infected cells presented almost the same number of dextran spots over the inspected area, and uptake was similar to that observed in non-infected cells (mock) (Figure 7B).

In contrast to the reduced dextran internalization, FM4-64 was highly endocytosed in cells infected by STM SL^WT^ compared to non-infected cells (Figures 7C, D). Non-infected cells showed homogenous cytoplasmic membrane labeling by FM4-64 on the apical side (Figure 7C), while in infected cells, FM4-64 signals appeared below the apical membrane and separated from apical F-actin. The FM4-64 signals from infected and mock-infected cells were quantified, indicating that higher levels of intracellular dye in STM SL^WT^-infected cells were higher than measured for mock-infected cells (Figure 7D).

These results demonstrate that after internalization of STM and apparition of RA, PEC loses the ability to resorb material from the luminal space. This effect is mediated by the translocation of SopE, but not SopE2, since cells infected by strains lacking *sopE2* could resorb apical markers, as shown in Figure 7C. Therefore, SopE dominantly affects the physiological resorption of host cells, likely due to its specific effect on Rac1 (Friebel et al., 2001). Although other strains expressing only SopE2, which acts on Cdc42, could efface MV and induce RA, they failed to abrogate this cellular function in the PEC infection model.

### Invasion by STM induces the recruitment of ezrin to *Salmonella*-containing vacuoles

**Figure 8:**
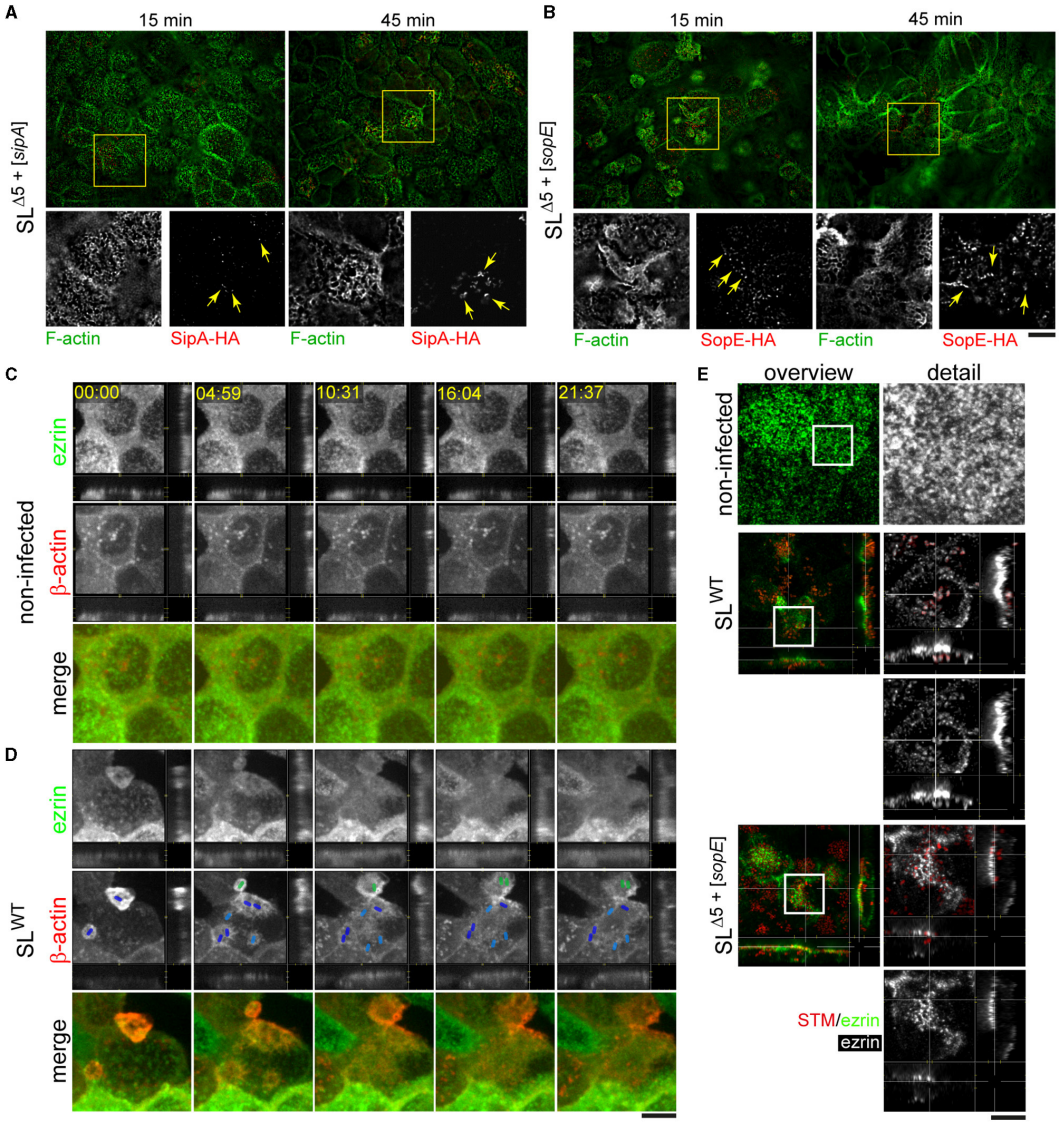
STM induces RA by translocation of SopE and SipA and recruits ezrin to the nascent SCV. PEC were infected with STM SL^Δ5+[*sipA*::*HA*]^ or SL^Δ5+[*sopE*::*HA*]^ (red) as described in Figure 1B for 15 or 45 min. After fixation and permeabilization, immunostaining for translocated effector proteins with anti-HA antibody (red) was performed, and F-actin was labeled with phalloidin (green) **(A, B)**. Alternatively, infected cells were immunolabeled with anti-ezrin antibody (green), and F-actin was stained by phalloidin (red) **(C, D)**. **(A)** Formation of RA is triggered by SipA only independent of SopE. SipA reorganizes the actin cytoskeleton into RA structures. **(B)** The formation of RA is triggered by SopE only independently of SipA. Both effector proteins strongly localize at the actin filaments at 45 min p.i. Yellow arrows indicated effector proteins SipA **(A)** or SopE **(B)** in the apical portion of MDCK cells. **(C, D)** Ezrin is delocalized after engulfment of STM. MDCK cells transfected with ezrin-eGFP and β-actin-RFP were infected with STM SL^WT^ (not visible, positions indicated by colored rod symbols: dark blue, initial invading STM; light blue, subsequent invading STM; green, STM invading another host cell). See corresponding Supplementary Movie 2 for the time-lapse sequence. **(E)** STM recruits ezrin for the early SCV after the invasion of PEC. MDCK cells were infected with STM SL^WT^ or SL^Δ5+[*sopE*]^. Images are representative of at least three independent experiments. Scale bars, 15 μm and 5 μm **(A, B)** for overview and detail, respectively; 5 μm **(C)**; 20 and 10 μm **(D)** for overview and detail, respectively. Timestamp, min:sec.

Our results demonstrate that only cells highly infected by strains lacking SopE exhibited RA, which diminished after gentamicin treatment. Furthermore, the quantification of the actin polymerization rate was only altered in those cells infected by SL^WT^ or NCTC^WT^ strains. The apical endocytosis of infected cells was altered by the translocation of SopE but not SopE2. These observations indicate that there may be further SPI1-T3SS effector protein functions necessary to cause actin rearrangements in the absence of SopE and SopE2 but are unable to alter apical endocytosis. Our previous work has already demonstrated that SipA can induce actin polymerization by STM strains lacking SopE and SopE2 (Schlumberger and Hardt, 2005; Schlumberger et al., 2007; Felipe-Lopez et al., 2023). Therefore, we used the same reductionist approach to test if SipA induces the formation of RA. We infected cells for 15 or 45 min with STM SL^Δ5+[*sipA*]^ or SL^Δ5+[*sopE*]^, and both effector proteins and F-actin were stained (Figures 8A, B). Micrographs acquired by total internal reflection microscopy (TIRF) indicated that effector proteins were localized in the F-actin-rich apical area of PEC and at 45 min p.i. sufficient amounts of effector protein accumulated to compare the distribution. While SopE was decorating RA structures at larger areas of the apical side of cells (Figure 8B), SipA was highly concentrated in small regions with F-actin (Figure 8A). The distinct distribution was in line with the observed membrane association of SipA close to the SPI1-T3SS translocon and the cytosolic distribution of SopE. The apparent reticular structure was associated with the bacteria residing (not visible) on the apical side of host cells, which continuously translocate SipA, as observed in Figure 8A (detail, 45 min).

Because STM induces massive brush border effacement and diminishes the apical resorption of fluid tracers, we considered that microvillar proteins might be lost after complete MV effacement, which would explain the origin of RA and reduced endocytic uptake. Therefore, we followed the spatial distribution of ezrin after MV effacement. MDCK cells transfected for the expression of ezrin-GFP and β-actin-RFP were infected by STM SL^WT^ (Figure 8C; Supplementary Movie 2) since we observed the most severe phenotypes with this strain. Ezrin clearly localized in MV in non-infected cells or in cells prior to infection. After STM triggered ruffle formation and MV effacement was initiated, ezrin was lost from the MV and recruited to ruffles. However, once STM was completely engulfed, cells did not recover ezrin at the apical membrane. Instead, the signal of ezrin mainly remained in the cytoplasm at the apical side, as images from lateral projections from LCI sequences indicate (Figure 8D). In contrast, non-infected cells showed no changes in the localization of ezrin over the acquisition period (Figure 8C). These results may indicate the recruitment of ezrin to nascent SCV after complete engulfment. To address this hypothesis, MDCK cells were infected for 45 min and stained with antibodies against ezrin. Most ezrin signals were concentrated at nascent SCV harboring STM SL^WT^ (Figure 8E), compared to non-infected cells, which maintained the ezrin at the apical side of the cell. To evaluate whether the recruitment of ezrin is caused by SopE, we used STM SL^Δ5+[*sopE*]^, only translocating SopE, but able to invade PEC and to remodel their actin cytoskeleton (Felipe-Lopez et al., 2023). In cells infected by STM SL^Δ5+[*sopE*]^, ezrin was delocalized from the apical membrane as observed in STM SL^WT^-infected cells; however, no prominent association with nascent SCV was observed (Figure 8E).

These data demonstrate that SopE mainly controls actin remodeling after complete engulfment by STM. Although SopE-deficient strains could cause MV effacement and induce RA by translocating SipA, these morphological changes were not sufficient to alter the physiological resorption by PEC or to modify actin polymerization to the extent observed in SopE by a single bacterium. The delocalization of ezrin caused by SopE-induced actin rearrangements is an example of the loss of microvillar proteins during the invasion of STM, which might indirectly lead to reduced resorption by PEC.

## Discussion

The effacement of MV occurs during infection by various enteric pathogens such as EPEC, *Helicobacter pylori*, or *Citrobacter rodentium*. In contrast to the local destruction of MV by these pathogens, STM invasion causes the complete collapse of MV in PEC, such as enterocytes (Takeuchi, 1967). We previously described that the sole translocation of SopE into host cells caused MV effacement, induced the formation of RA, and mainly mediated the invasion of PEC (Felipe-Lopez et al., 2023). In this study, we additionally found that SopE is a key factor for disturbing resorption by PEC. Yet, strains lacking SopE were still able to efface MV by the accumulation of bacterial cells on the apical side of PEC over prolonged periods of infection. In both cases, the internalization of STM culminated in the appearance of RA on the apical side of PEC. RA remained even after the complete internalization of STM SL^WT^ or NCTC^WT^, but disappeared in cells infected by STM SL^Δ*sopE*^ or NCTC^Δ*sopE2*^, permitting host cells to partially recover MV after complete STM internalization. Although SopE-deficient strains also efface MV and create RA, their infected cells are still able to endocytose dextran.

Our data demonstrate that reduced invasion due to the absence of SopE can be compensated by prolonged periods of STM exposure that increase the opportunity for multiple STM to interact with host cells and to form bacterial clusters on the PEC apical side (see Figures 1B–F). Our experimental setup is similar to that published by Lorkowski et al. (2014); thus, the accumulation of bacteria is the result of the cooperativity of STM infection, allowing other bacteria to internalize once a “starter” STM cell has triggered membrane ruffles (Misselwitz et al., 2012; Lorkowski et al., 2014). In contrast to STM SL^WT^, STM NCTC^WT^ did not trigger large ruffles; instead, it formed microcolonies that concluded with the loss of brush border and formation of RA, which completely replaced the brush border architecture.

While all *S. enterica* strains harbor *sopE2*, only a subset additionally possesses *sopE* due to lysogenic conversion through infection by *sopE* phage (Mirold et al., 1999). Previous research showed that several virulence traits of STM are associated with the function of SopE, such as intestinal inflammation due to caspase-1 activation (Muller et al., 2009) and activation of NOD1 signaling (Keestra et al., 2013). By comparing intracellular phenotypes of STM strains with and without SopE, we recently demonstrated that the presence of SopE leads to increased invasion, damage of the nascent SCV, and escape of STM into host cell cytosol (Röder and Hensel, 2020). Sensing cytosolic STM by the NAIP/NLRC4 inflammasome results in the expulsion of infected PEC into the intestinal lumen (Chong et al., 2021; Fattinger et al., 2021a), and this increases shedding and the further spread of STM. The data reported here add reduced resorption by PEC as a further pathogenic consequence of the presence of SopE in STM strains. Therefore, SopE can be considered as a factor increasing the severity of STM intestinal infections.

MV effacement is caused by bacterial accumulation and RA formation, but capability in endocytosis suggests that a third effector protein is involved in the absence of SopE and SopE2. Indeed, only SipA participated in the formation of RA, as our reductionist approach revealed. We previously demonstrated that SipA, SopE, and SopE2 independently mediate ruffle formation or discrete actin polymerization, leading to the invasion of PEC (Felipe-Lopez et al., 2023). Our observations are supported by results from murine infection models (Zhang et al., 2018; Fattinger et al., 2020). STM can discretely penetrate enterocytes exclusively by the translocation of SipA in the absence of SopE, SopE2, or both SopE and SopE2. These *in vivo* observations support our data showing that RA appears after infection by STM strains only expressing SipA. In our model, the absence of SopE and SopE2 did not alter the endocytosis of PEC and SL^Δ*sopE*^, SL^Δ*sopEsopE2*^, or NCTC^Δ*sopE2*^still invaded if sufficiently long interaction with host cells was allowed. Contrary to our *in vitro* observations, MV are not effaced *in vivo* by SipA-mediated invasion. These observations would suggest that SipA-mediated invasion of STM may reduce the damage to the intestinal epithelium. Nevertheless, SipA and ezrin mediate the recruitment of neutrophils (Agbor et al., 2011), and inflammation of epithelial tissue is caused by strains expressing only SipA in the ileal loop model (Zhang et al., 2002).

The formation of MV or actin networks is directly proportional to the density of proteins recruited to the apical side of the cell, including bundling, anchoring proteins, and actin (Gov, 2006). Once this group of proteins localizes at the apical side of PEC, actin polymerization and an assembly of bundles originate a protrusion force that first creates actin networks. Further, actin polymerization by treadmilling creates new MV on the apical surface (Gaeta et al., 2021). In contrast, if actin polymerization is disturbed or membrane anchoring fails, then no further bundling proteins are recruited, as we observed with infection of STM.

Hence, in our model, actin polymerization is redirected to ruffle formation by the activity of SipA, SopE, and/or SopE2; the further disorganization of ezrin and depolymerization of actin by villin (Lhocine et al., 2015; Felipe-Lopez et al., 2023) would explain that infected host cells fail to restore their brush border architecture. Instead, the apical surface remains as a reticular structure until the action of virulence proteins ceases, as we observed in those cells treated with gentamicin. Further published evidence supports this model. MV elongation and stabilization depended on the binding and capping functions of EPS8, villin, epsin, and fimbrin (Meenderink et al., 2019; Gaeta et al., 2021). Indeed, PEC of ezrin knockout mice only developed short MV, a disorganized terminal web (Saotome et al., 2004), and showed altered localization of apical transporters (Engevik and Goldenring, 2018). In mice lacking either villin, epsin, fimbrin, or a combination of these three proteins, MV were specifically reduced in amount and developed only short-length structures (Revenu et al., 2012). In yeast, fimbrin contributes to the breaching of F-actin filaments depending on the fimbrin concentration (Laporte et al., 2012). Moreover, the expression of EPS8 also regulates the length of MV in porcine kidney cell lines. Therefore, at low concentrations of fimbrin, disassociation of MV proteins such as ezrin, villin, or ESP8, actin filaments form highly ramified structures as we observed in this study, and supported by our previous work (Felipe-Lopez et al., 2023).

**Figure 9:**
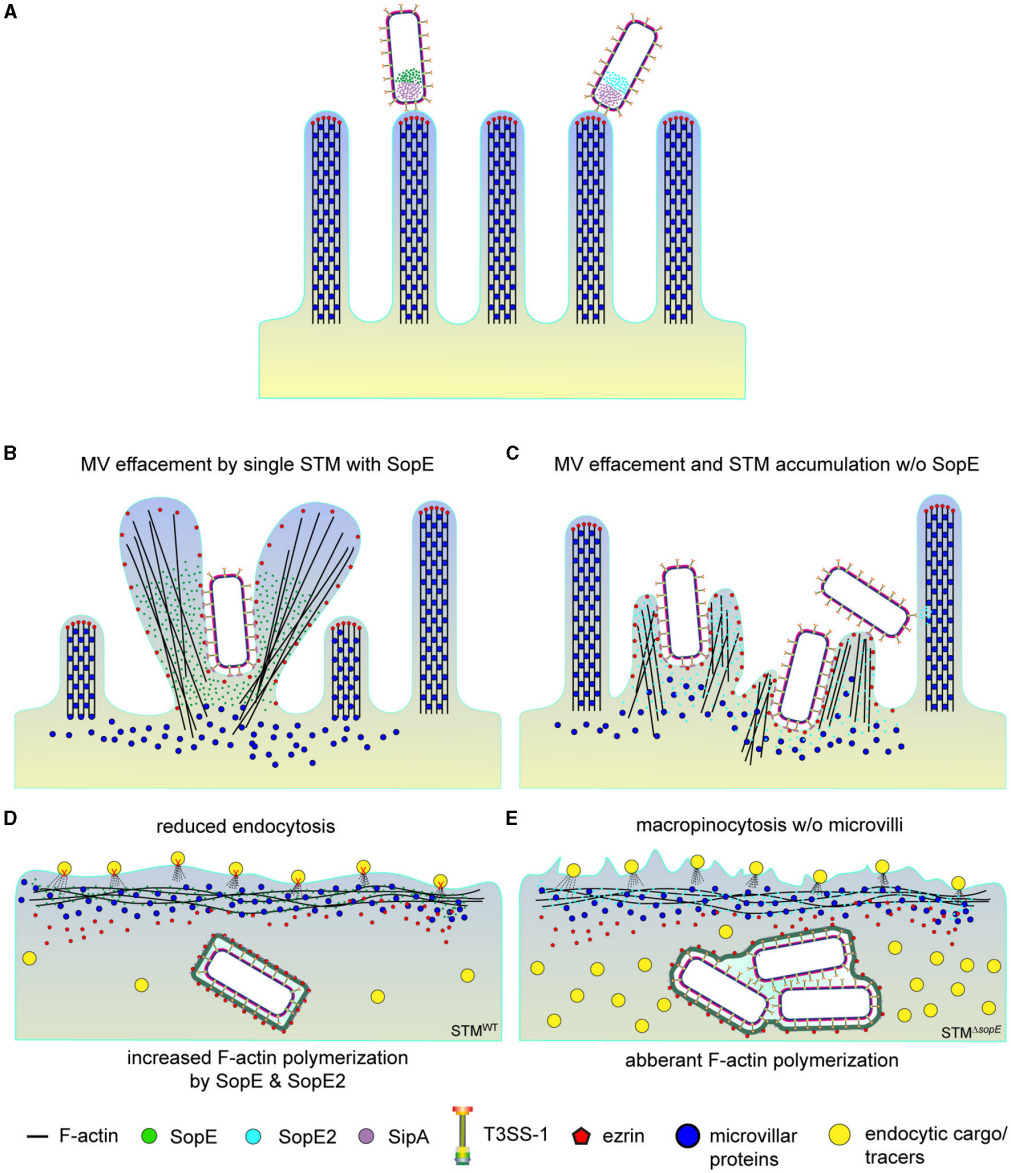
Destruction of the brush border by SPI1-T3SS effector SopE abolishes endocytosis of PEC. The model depicts the sequence of events observed during the STM invasion of PEC. **(A)** After adhesion, STM translocate SPI1-T3SS effector proteins into host cells. **(B)** SopE-induced MV effacement also causes delocalization of ezrin and probably other microvillar proteins affected, whereas **(C)** bacterial cells of STM lacking SopE accumulate to cause local actin reorganization and MV effacement. **(D)** Internalization of STM-secreting SopE concludes with RA formation (not displayed), recruitment of ezrin to the nascent SCV, and severe reduction of resorption of luminal content (tracers). Delocalization of ezrin and other microvillar proteins probably prevents recovery of MV but has minor effects on resorption since cells infected by SopE-negative STM **(E)** still endocytose dextran are representative endocytic cargo.

Based on our observations, we propose that loss of ezrin from MV during STM invasion hinders the anchoring of new F-actin filaments (Figure 9). In turn, this would avoid the formation of MV, as previously described (Gloerich et al., 2012; Solaymani-Mohammadi and Singer, 2013; Dhekne et al., 2014; Gaeta et al., 2021). Since actin polymerization required for MV formation is redirected to sites of STM invasion, other bundling proteins may not be properly recruited. Consequently, low concentrations of bundling proteins such as fimbrin (Revenu et al., 2012), ezrin (along with EPS8) (Gaeta et al., 2021), and villin (Meenderink et al., 2019; Felipe-Lopez et al., 2023) may only permit the formation of RA after the complete internalization of *Salmonella* (Figure 9C). The loss of ezrin from the apical side of PEC may be attributed to the degradation of phosphoinositide 4,5 bis-phosphate (PI-4,5P_2_) by SopE-activated phospholipase γ (PLC γ) and SopB (Terebiznik et al., 2002; Felipe-Lopez et al., 2023).

MV effacement and formation of RA have further consequences for PEC in this model. Cells infected by SopE-harboring STM lost MV and were deficient in dextran uptake from the luminal side. Previous observations with MDCK cells attributed the altered uptake of fluid tracers to the inhibition of members of the Src kinase pathway (Mettlen et al., 2006). These authors found that after Src kinase activation by decreasing the temperature from 40 to 34°C, cells formed ruffles, which enhanced the uptake of dextran. The uptake of dextran and holo-transferrin was blocked by inhibitors of F-actin formation, phospholipase, and PIP5K, which indicates the participation of active actin polymerization. In our model, the polymerization of actin in infected cells was 50% higher in zones with RA. Despite SopE2 also altering the actin polymerization, endocytosis in cells infected by STM only containing SopE2 was not altered. Therefore, the alteration of actin polymerization by SopE, but not SopE2, and loss of the absorptive surface of PEC are factors affecting the endocytosis of infected cells.

In contrast, we found that the membrane stain FM4-64 was highly adsorbed only in infected cells, where *Salmonella* secretes SopE and forms a macropinocytic cup. The accumulation of FM4-64 was previously associated with the production of phosphatidyl-inositol 3,4,5, tri-phosphate (PI-3,4,5P_3_) in macropinocytic cups of macrophages stimulated with M-CSF (Yoshida et al., 2009). PI-3,4,5P_3_ is a product of PI3K, a kinase downstream of Rac1. These observations relate to infection by STM expressing SopE; since SopE is a GEF for Rac1, consequently, constant activation of Rac1 via SopE may have further effects on the PI pool at the plasma membrane, which increase during the formation of macropinocytic cups of STM internalization. We speculate that the strong activation of Rac1 by SopE may additionally be the cause of the high recruitment of other proteins, such as Rab5, necessary for the closure of the macropinocytic cups. If so, the amount of Rab5 and other proteins necessary for proper macropinocytosis could be exhausted during STM infection, thus preventing the resorption of luminal content such as dextran. This result also suggests that the selective action of SopE2 on Cdc42 would not affect other proteins necessary for physiological endocytosis.

Similar to our infection model of *Salmonella* in PEC, infection of C2BBe1 cells by EPEC also culminated in a reduction of the uptake of substrates from the lumen, accompanied by the loss of function of the sodium/D-glucose cotransporter SGLT (Dean et al., 2006). These phenotypes mainly correlated to the infection doses in the experiments and translocation of EPEC effector proteins Map, EspF, Tir, and adhesin Eae. These proteins are necessary to remodel the F-actin in MV since Map binds to the PDZ domain, which interferes with the association of the NHERF transporters to the F-actin cytoskeleton in MV. Its action is enhanced by a cooperative mechanism of the proteins NheI and EspI (Martinez et al., 2010) and the NleH effector protein of EPEC targeting EPS8 to reorganize the apical surface of the host cells to form microcolonies (Lhocine et al., 2015).

Our observations also suggest that effector proteins may not have redundant virulence functions in the host cells but rather cooperatively act to permit a proper internalization of STM into host cells. Another interesting point for further investigation is the reduction of virulence to one or two effector proteins using reduced bacterial infecting doses and long incubation periods for bacterial internalization. Using this setup, we observed no extreme alterations in host cells. Whether this may be sufficient to penetrate enterocytes and disseminate into lymph organs without causing inflammation is still open since SipA triggers neutrophils into the infection area *in vivo* and *in vitro* (Zhang et al., 2002; Agbor et al., 2011). It would be interesting to know whether this inflammation causes erosion of the epithelial layer and confines intestinal infection.

Altogether, our data demonstrate that translocation of SopE and SipA dominantly induces the formation of RA at the apical side of host cells, which is a consequence of the loss of ezrin and probably other microvillar proteins after internalization of *Salmonella*. Furthermore, loss of MV and maintenance of RA via SopE abrogate endocytosis of infected PEC. These physiological alterations of the apical side of PEC and the reduction of the uptake function seem to be a common result of infections by intestinal pathogens. This points out that the organization of the actin and endocytic capacity are highly interdependent processes and are regulated by the same family of proteins in MV, which are directly or indirectly manipulated by bacterial virulence proteins. Further investigations are needed to identify the precise molecular mechanisms behind the alteration of endocytic capacity caused by infection with intestinal pathogens and the physiological consequences.

## Materials and methods

### Bacterial strains, construction of mutant strains, and plasmids for complementation

*Salmonella enterica* serovar Typhimurium strain SL1344 (SL^WT^) and NCTC 12023 (NCTC^WT^) were used as wild-type strains, and their isogenic mutant strains were employed throughout the experiments described in this study (see Table 1). Gene deletion strains were generated by the insertion of an *aph* cassette into specific target genes. Mutations were generated in NCTC12023 by red-mediated recombination (Datsenko and Wanner, 2000) using pKD13 as a template and primers specified in Table 2. If required, mutant alleles were transferred to the SL1344 strain background by P22 transduction as previously described (Felipe-Lopez et al., 2023).

**Table 1:** Bacterial strains used in this study.

**Designation**	**Relevant characteristics**	**Reference**
***S. enterica*** **serovar Typhimurium SL1344 strains**		
SL1344	wild type, Sm^R^	Lab stock
M712	Δ*sipA sopA sopB sopE sopE2*	Ehrbar et al., 2004
M1318	Δ*sopE*	(Hardt Lab)
SB856	Δ*sopE*::*aphT*, Km^R^	Hardt et al., 1998
MvP1459	Δ*sopE2*::*aph*, Km^R^	Felipe-Lopez et al., 2023
MvP1485	Δ**sopE*2::*aph**, Km^R^	This work
***S. enterica*** **serovar Typhimurium NCTC 12023 strains**		
NCTC12023	Wild type, Nal^S^	Lab stock
MvP1412	Δ*sopE2*::*aph*, Km^R^	This study

**Table 2:** Oligonucleotides used in this study.

**Designation**	**Sequence 5^′^-3^′^**
SopE2-Red-Del-For	aaagtgtagctatgcatagttatctaaaaggagaactaccgtgtaggctgga gctgcttc
SopE2-Red-Del-Rev	taattcatatggttaatagcactattgtatttactaccacatatgaatatcctc cttag
SopE2-Red-Check-For	ctaaaaggagaactaccgtg

### Cell lines and culture conditions

All cell lines employed in this study were cultured at 37°C in a humidified atmosphere containing 5% CO_2_. For invasion assays and microscopy analyses, MDCK clone Pf was used as a standard cell culture model, kindly provided by the Nephrology department of the University Hospital Erlangen, FAU Erlangen-Nürnberg. Confluent monolayers in 25 cm^2^ cell culture flasks were seeded each week in a new 25 cm^2^ flask with MEM supplemented with 1 × non-essential amino acids (PAA, Germany), 10% inactivated fetal calf serum (FCS, Sigma, Germany) and 1 × Glutamax (PAA, Germany). Changes in the trans-epithelial electrical resistance (TEER) due to the infection of *Salmonella* were monitored using the cell line Caco-2 BBe1, which is a derivate of Caco-2 cells (ATCC CRL-2102). These cells were cultured in DMEM high glucose without pyruvate (PAA, Germany), containing Glutamax, 10% FCS, and 2.5 μg × mL^−1^ human holo-transferrin (Sigma-Aldrich, Germany). Cells were seeded at 10^5^ cells per 12 mm polycarbonate filter insert (0.4 μm pore size, Millipore, Germany). TEER was measured every third day with a platinum electrode and an Ohm meter EVOM (World Precision Instruments, USA). Cells were cultured until a TEER of 500–700 Ω per well was observed, usually 10–15 d. For cultivation, media were supplemented with penicillin/streptomycin (PAA, Germany). The medium was changed every third day.

### Invasion assays

Five days prior to infection, MDCK cells were seeded at 1 × 10^5^ cells per well in 24-well plates (Nunc, Denmark). At least 4 h before infection, the medium was substituted with a medium without antibiotics. Bacterial strains were inoculated in LB and incubated overnight at 37°C with continuous aeration. Cultures were diluted 1:31 in fresh LB and incubated for another 4 h in glass test tubes in a roller drum. Next, the optical density of each culture was measured and adjusted in 1 ml MEM to OD_600_ = 0.2 (estimated 3 × 10^8^ bacteria × mL^−1^) to generate a master mix. Cells were infected at multiplicity of infection (MOI) as indicated in subsequent sections, and assays were performed in triplicates for each strain and infection condition. After incubation at indicated periods of time for invasion, cells were washed three times with PBS. Next, fresh medium with gentamicin at 100 μg × mL^−1^ was added for 1 h. Finally, infected cells were washed five times with PBS and lysed with 0.5% deoxycholic acid for 10 min. Lysates were diluted and plated onto Mueller Hinton II agar (BD, Germany) plates with an Eddy Jet spiral platting instrument (IUL Instruments, Barcelona). Plates were incubated at 37°C overnight, and colony-forming units (CFU) were counted. For infection of monolayers on filter inserts, bacterial strains were added at MOI 50, and TEER was recorded every 20 min over a period of 2 h. Finally, cells were washed three times with pre-warmed PBS and fixed with methanol at −20°C overnight.

### Immunostaining

For imaging, bacterial strains harbored pWGR435 or pFPV-mCherry for constitutive expression of mTagRFP or mCherry, respectively, under the control of P_*rpsM*_ (plasmids are listed in Table 3). The same amount of cells as for invasion (see above) were seeded on coverslips and infected in duplicates at MOI 50 with a master mix of the respective STM strain. At various time points after infection, infection was stopped by washing cells with PBS four times. Cells were immediately fixed with pre-warmed 3% PFA in PBS for 1 h at 37°C. After fixation or any subsequent incubation with fluorescent dyes or antibodies, cells were washed three times with PBS at 37°C. Fixed cells were permeabilized by incubation for 15 min at 37°C with 0.5% Triton X-100 (Sigma-Aldrich, Germany) in a blocking solution consisting of 2% BSA (Biomol, Germany) and 2% goat serum (Gibco, Germany) in PBS in a humid chamber. Antibodies were diluted in a blocking solution, and incubations were performed at 37°C in a humid chamber. Rabbit anti-ezrin (Dianova, Germany) was diluted 1:100 and incubated for 1 h. Actin-stain 488-conjugated phalloidin (Cytoskeleton, France) was added at 1:200 dilution and incubated for 45 min at 37°C. Coverslips were then mounted on glass slides with Fluoroprep (Biomerieux, France), sealed with Entellan (Merck, Germany), and kept in the dark at 4°C.

**Table 3:** Plasmids used in this study.

**Designation**	**Relevant characteristics**	**References**
pWSK29	Low copy number, Carb^R^	Wang and Kushner, 1991
p4040	pWSK29 *P_*sicA*_::sipA*::HA	Felipe-Lopez et al., 2023
p4043	pWSK29 *P_*sopE*_*::*sopE*::HA	Felipe-Lopez et al., 2023
p4044	pWSK29 *P_*sopE*2_*::*sopE2*::HA	Felipe-Lopez et al., 2023
pKD4	Vector for *aph* cassette	Datsenko and Wanner, 2000
pKD13	Vector for *aph* cassette	Datsenko and Wanner, 2000
pKD46	λ Red expression	Datsenko and Wanner, 2000
pCP20	FLP expression	Datsenko and Wanner, 2000
p3589	*P_*rpsM*_*::mCherry in pETcoco, Cm^R^	This work
pWRG439	P*_*rpsM*_*::mTagRFP in pFPV25.1, Carb^R^	Roman G. Gerlach

### Apical uptake of fluorescent fluid tracers

Fluorescent fluid tracers were deployed to evaluate the uptake at the apical side of MDCK cells after infection and destruction of the brush border. Cells were infected as described for invasion experiments (see above). After 1 h of gentamicin treatment, cells were washed three times with pre-warmed PBS. Next, cells were pulsed with 250 μg × mL^−1^ dextran Alexa-568 10,000 FX, or 50 μg × mL^−1^ of the membrane dye FM4-64FX (Life Technologies, Netherlands). Both fluid tracers were mixed in MEM, 30 mM HEPES, pH 7.4 w/o phenol red, and w/o sodium bicarbonate. Cells were then incubated for 30 min at 37°C at 5% CO_2_. Finally, tracers were removed from the cells by washing them three times with pre-warmed PBS. They were then fixed with 3% PFA and further processed for microscopy as described above with additional staining of DNA by adding DAPI at 1:1,000 to Fluoroprep.

### Microscopy and live-cell imaging

Images from the fixed samples were acquired with a DMI 6000 SP5 II confocal laser-scanning microscope (Leica Microsystems Wetzlar, Germany). They were acquired with a 100x objective with a numerical aperture of 1.44. The pinhole was adjusted to 1 Airy unit for all acquisitions, and the pixel size of the images was 70.85 × 70.85, with a bit depth of 16 bits. Z-slice thickness was adjusted to 0.12 μm using the Nyquist theorem. The Ar 488 nm laser line was used for Alexa488-conjugated antibodies and eGFP. The HeNe 543 nm laser line was used for the excitation of Alexa568-conjugated antibodies and mCherry or mTagRFP. Images were acquired with Leica Acquisition Software V. 2.3.6. and further processed with Imaris V. 7.6.1 (BitPlane, Switzerland) and FIJI (Max-Planck Institute for Cell Biology, Dresden, Germany).

Live-cell imaging (LCI) was performed using Lifeact-EGFP MDCK cells, which are described before (Felipe-Lopez et al., 2023), and mCherry-, or mTagRFP-expressing *Salmonella* strains. LCI was performed using a CellObserver microscopy system (Zeiss, Germany) coupled to a Yokogawa spinning disc unit. Images were acquired for 120 min shortly after infection at maximal speed with intervals of 100–200 ms at distances between *Z* planes of 0.30–0.35 μm. LCI was performed with a water immersion objective with a numerical aperture of 1.333. The acquisition was performed with either a cooled CCD camera (CoolSNAP HQ^2^, Photometrics) with a chip of 1,040 × 1,392 pixels for high spatial resolution or an EM-CCD camera (Evolve, Photometrics) with a chip of 512 × 512 pixels for high sensitivity and FRAP analysis. The acquisition and processing of the time-lapse images were performed with ZEN 2012. Images from CLSM or SD were deconvolved with Huygens V.4.2 using a theoretical PSF. Bleaching and *Z*-drift were also corrected with Huygens. Further processing and movie export were performed with Imaris 7.6.1.

### Quantification of endocytosed fluorescent fluid tracers

Fixed samples of infected MDCK cells fed with dextran Alexa568 10 000 FX were observed by laser confocal-spinning disc microscopy with an EmCCD camera (see above). DAPI-stained structures were excited by a 405 nm laser line; F-actin stained with phalloidin Actin-stain 488 was excited with the laser line 488 nm; finally, dextran-stained samples were excited with a laser line 561. Images were acquired with a 63x oil immersion objective with a refraction index of 1.51 and a numerical aperture of 1.44 through the Z axis with intervals of 0.12 μm between each slice. Images were further deconvolved using Huygens 4.2. for the detection of dextran in all other cells. Then, restored images were visualized in Imaris V.7.6.1. For quantification, dextran spots of the non-infected cells and non-stained cells were set up at a lower threshold for processing the rest of the infected samples. The minor diameter of dextran spots was defined to be ~ 0.5 μm. Quantification of dextran was then performed using the “Spot” function of Imaris, briefly: a region of interest of 45 × 45 pixels, which represents approximately one cell, was set. Next, the spots with a diameter of ~0.5 μm were recognized by their maximal intensity signal. The threshold values from the emission spectrum of each sample were then adjusted from 450 to 10,000 voxels from a total emission spectrum of 16,000 voxels. Finally, the whole observation field was processed with these parameters. The average of the total number of spots per field was calculated and plotted as indicated in the Results section.

Images from samples stained with the membrane fluorescent dye FM4-64FX were acquired by confocal laser-scanning microscopy (see below) with the XZY mode. This mode allows the acquisition of a vertical section of the sample w/o *Z*-slices. Therefore, the observation of the distribution of the membrane dye throughout the cytoplasmic membrane was possible. Samples were observed with a 100x-objective with a numerical aperture of 1.44 and a refraction index of 1.51. Image resolution was 1,024 × 1,024 pixels from a field size of 45 × 45 μm with a bit depth of 8 bits. The thickness of each slice in the *Y*-axis was 0.12 μm. FM4-64 or phalloidin Actin-stain 488 were excited by the Ar 488 nm laser line. Images were further processed with FIJI. A profile line was drawn at the apical side of each section. The total signal from the apical side of the section was added, and the average of this signal from several fields was calculated and plotted, as shown in the results section.

### Fluorescence recovery after photobleaching in infected MDCK cells

To evaluate the polymerization of F-actin to RA, fluorescence recovery after photobleaching (FRAP) was performed in cells infected by *Salmonella*. MDCK Lifeact-EGFP cells were seeded on treated chamber slides (Ibidi, Germany) at 25,000 cells per well. After 5 days, the medium was substituted with MEM 30 mM HEPES pH 7.4 w/o sodium bicarbonate and phenol red. Then, cells were infected by *Salmonella* at MOI 25 as described for invasion experiments. At 25 min p.i., cells were washed with a pre-warmed MEM medium to avoid alterations of any cellular structures such as MV or RA. Cells were further incubated with MEM as described above and observed by SD microscopy.

Image acquisition was performed at the apical side of the infected cells with a frame rate of 100 ms per frame per channel. The 488 nm and 561 nm laser lines were set to 2 and 5% intensity, respectively. The electrical gain of the EmCCD camera was set to 100 in both channels. Before bleaching, 50 frames were acquired. A bleaching pulse was performed for 500 ms with a 488 nm laser line at transmission of 100% using a pre-established mask of 5 μm. Signal recovery was quantified during 200 frames after the bleaching pulse. Results from the FRAP signal were plotted as relative signals over time in seconds. The recovery constant was calculated from the bleaching point until the next 200 frames by the method described by Ishikawa-Ankerhold et al. (2012). Results were plotted as described above.

### Atomic force microscopy

To observe topological changes in cell surface as a consequence of STM*-*induced cytoskeletal remodeling, AFM measurements were conducted using the NanoWizard II AFM system (JPK Instruments AG, Berlin, Germany). High-resolution surface images were acquired by operating the AFM under ambient conditions in soft contact mode using silicon nitride AFM probes with a nominal force constant of 0.06 N/m (SiNi, Budget Sensors, Wetzlar, Germany). Samples were prepared as described above. For each sample, topographic overview images with a 90 × 90 μm scan area were taken before zoom-ins were generated. All images were polynomially fitted and unsharpened mask filtered using JPK data processing software (JPK Instruments AG). Presented images are 3D projections of the height profiles, tilted 12° in X direction.

## Data availability statement

The raw data supporting the conclusions of this article will be made available by the authors, without undue reservation.

## Author contributions

AF-L: Conceptualization, Data curation, Investigation, Project administration, Validation, Visualization, Writing – original draft, Writing – review & editing, Formal analysis, Methodology, Resources. NH: Conceptualization, Data curation, Formal analysis, Investigation, Methodology, Resources, Validation, Visualization, Writing – original draft. MH: Conceptualization, Data curation, Investigation, Validation, Visualization, Writing – original draft, Funding acquisition, Project administration, Supervision, Writing – review & editing.

## Supplementary material

Supplementary Figure 1 Manipulation of the apical side of PEC and F-actin cytoskeleton by STM. Previously reported analyses of the apical side of PEC during STM adhesion and invasion were compiled. **(A)** MDCK cells are infected by STM WT, fixed, and prepared for scanning EM. STM is pseudo-colored red. Modified from Gerlach et al. (2008). **(B)** MDCK cells were infected by STM WT expressing mCherry (red). At indicated time points, p.i. were fixed and labeled for F-actin (white). MV, microvilli; R, membrane ruffle; RA reticular F-actin. Arrowheads indicate RA. Modified from Lorkowski et al. (2014). **(C)** AFM analyses of surface topologies of STM-infected MDCK at indicated time points p.i. modified from Lorkowski et al. (2014). **(D)** Live-cell correlative live-cell scanning electron microscopy of MDCK monolayers expressing Lifeact (green in inserts), infected by STM WT (red in inserts). Arrowhead indicates RA. Modified from Kommnick et al. (2019). **(E)** CLSM and AFM of MDCK Lifeact-GFP cells apical side after infection by STM WT. The arrowhead indicates MV, R, or RA as indicated. Modified from Felipe-Lopez et al. (2023). For full experimental details, see the respective references. Timestamp min:sec. Scale bars, 10 μm (**A**, Left**, B, E**), 1 μm (**A** mid and right, **D**).

Supplementary Movie 1 STM destroys the brush border architecture of PEC and induces the formation of reticular F-actin. MDCK Lifeact-eGFP cells (gray) were infected with STM SL^WT^, SL^Δ*sopE*^, NCTC^WT^, or NCTC^Δ*sopE2*^. Bacterial strains were expressing mTagRFP or mCherry. Time-lapse series of LCI were recorded for 110 min. Timestamp, h:min:sec.ms. Scale bar, 5 μm. The movie corresponds to Figure 3.

Supplementary Movie 2 STM recruits ezrin to the nascent SCV. MDCK cells expressing GFP-ezrin (green) and β-actin-RFP (red) were infected with STM SL^WT^ (not visible), and a time-lapse series of LCI was recorded for 35 min. Timestamp, h:min:sec:ms. Scale bar, 5 μm. The movie corresponds to Figures 8C, D.
