# Supplementary figures and images for: Destruction of the brush border by Salmonella enterica sv. Typhimurium subverts resorption by polarized epithelial cells
Source: Front Microbiol. 2024 Jun 4;15:1329798. doi: 10.3389/fmicb.2024.1329798 (PMC11183102; doi:10.3389/fmicb.2024.1329798)

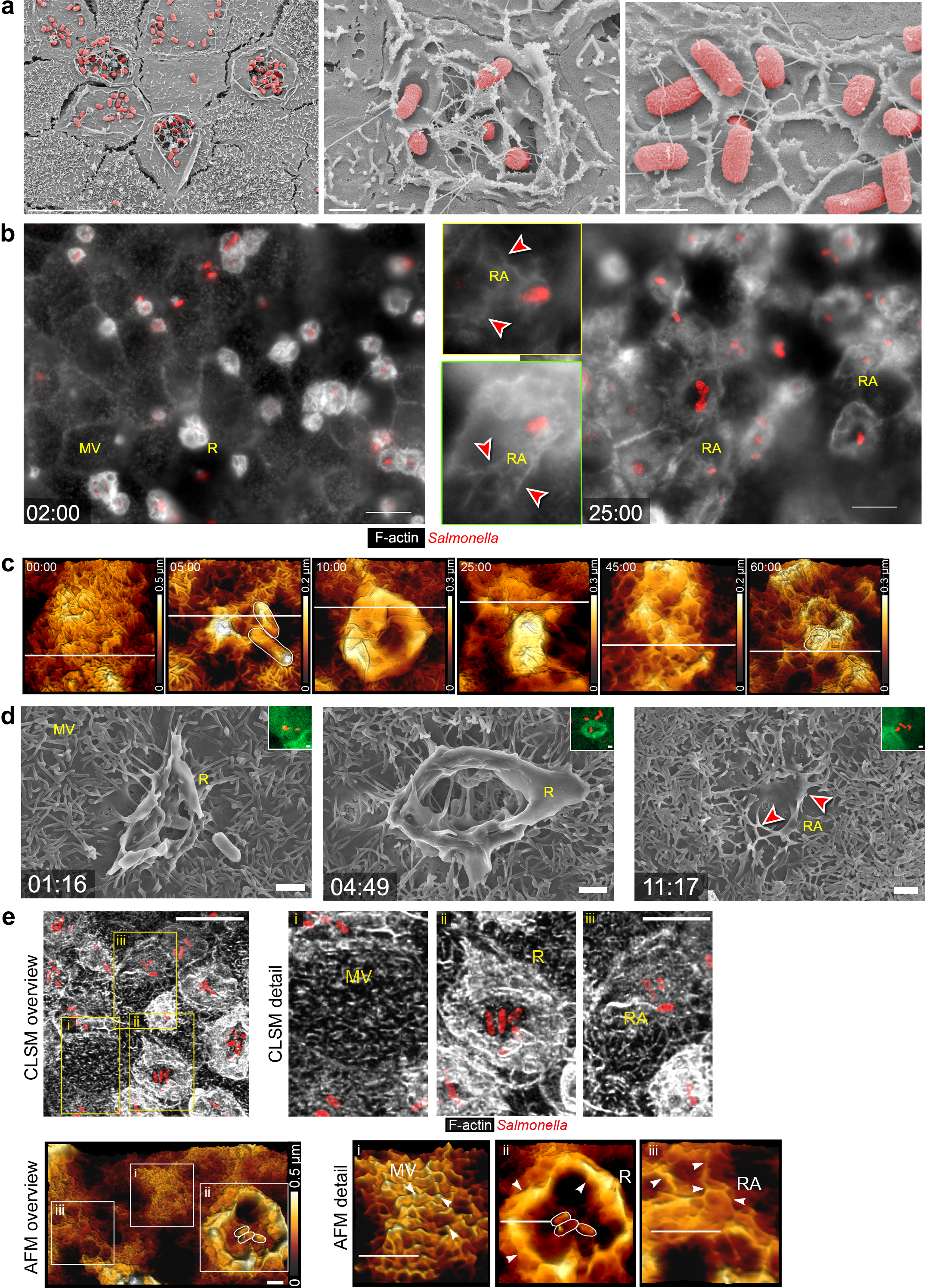

Supplement: Supplementary Figure 1 — Manipulation of the apical side of PEC and F-actin cytoskeleton by STM. Previously reported analyses of the apical side of PEC during STM adhesion and invasion were compiled. (A) MDCK cells are infected by STM WT, fixed, and prepared for scanning EM. STM is pseudo-colored red. Modified from Gerlach et al. (2008). (B) MDCK cells were infected by STM WT expressing mCherry (red). At indicated time points, p.i. were fixed and labeled for F-actin (white). MV, microvilli; R, membrane ruffle; RA reticular F-actin. Arrowheads indicate RA. Modified from Lorkowski et al. (2014). (C) AFM analyses of surface topologies of STM-infected MDCK at indicated time points p.i. modified from Lorkowski et al. (2014). (D) Live-cell correlative live-cell scanning electron microscopy of MDCK monolayers expressing Lifeact (green in inserts), infected by STM WT (red in inserts). Arrowhead indicates RA. Modified from Kommnick et al. (2019). (E) CLSM and AFM of MDCK Lifeact-GFP cells apical side after infection by STM WT. The arrowhead indicates MV, R, or RA as indicated. Modified from Felipe-Lopez et al. (2023). For full experimental details, see the respective references. Timestamp min:sec. Scale bars, 10 μm (A, Left, B, E), 1 μm (A mid and right, D). [file Image_1.TIF]
